# Relationships between fungal diversity and fruit quality of Yuluxiang pear during low temperature storage

**DOI:** 10.3389/fmicb.2023.1132271

**Published:** 2023-03-24

**Authors:** Yaru Hou, Xiaoyu Zhang, Zhenfeng Gao, Tian Chen, Lixin Zhang

**Affiliations:** College of Food Science and Engineering, Shanxi Agricultural University, Taiyuan, China

**Keywords:** pear, high-throughput sequencing, fungal diversity, low temperature, diversity analysis

## Abstract

Postharvest decay is an urgent problem that affects the storage of pears. Low temperature storage is one of the most important methods to reduce the prevalence of fruit diseases during storage. In this study, the microbial diversity of postharvest Yuluxiang pear (*Pyrus × michauxii* “Yu Lu Xiang”) fruits stored at low temperature for different lengths of times was analyzed. Illumina MiSeq high-throughput sequencing was used to analyze the composition and diversity of fungal communities. The results showed that the fungi within fruit were classified into 6 phyla, 18 classes, 40 orders, 72 families, and 92 genera based on the 97% sequence similarity level. They belonged to 6 phyla, 18 classes, 40 orders, 72 families, and 92 genera. The highest richness of fungi was obtained after 30 d of treatment. The β-diversity index showed that the fungal community composition of these fruit was significantly different at the beginning of storage compared with the different timepoints of samples at low temperature during storage. The comparison of fungal composition at the phylum level indicated that Ascomycota was dominant in the different timepoints of samples at low temperature, while *Alternaria* was the primary fungus at the genus level. A correlation analysis was used to further explore the correlation between fungi and fruit firmness, titratable acid, and solid soluble contents at low temperatures during storage. *Aureobasidium* and *Didymella* positively correlated with the soluble solids and hardness. *Phoma* positively correlated with the titratable acid, and *Aspergillus* positively correlated with titratable acid and hardness. This study can guide the industrial production of Yulu pear and also provide a theoretical basis to prevent and control diseases during the storage period of Yulu pear.

## 1. Introduction

Yuluxiang pear (*Pyrus × michauxii* “Yu Lu Xiang”) is the primary fruit grown in orchards in Shanxi Province, China. It is also one of the few varieties that is grown independently of China. Experts in the national pear industry system recognize it as China’s No. 1 pear (Ma et al., 2019; Zhang et al., 2020). The Yuluxiang pear has an important place in reorganizing the planting industry structure in Shanxi Province and serving as a strategic pillar industry to decrease the poverty of growers. However, owing to its thin peel and natural richness in nutrients, some types of pathogenic fungi and opportunistic pathogens can attach to its surface, and diseases, such as mold, can occur owing to improper temperature and humidity settings during the harvesting and storage processes (Sun et al., 2017). During the process of transportation and low temperature storage, fungal infection can cause common diseases, such as pear pulp browning disease, brown rot, and soft rot among others (Yu et al., 2012; Li et al., 2016). These conditions seriously affect the commodity value of fruits. Thus, the study of fungal diversity of fruits under low temperature storage has become an important issue.

In recent years, with the rapid development of molecular biology techniques and the popularization of extensive data analyses, high-throughput sequencing (HTS) technology is becoming an indispensable tool to study microbial community structure and composition (Xu et al., 2016; Dai et al., 2019; Abdel-Wahab et al., 2021). It provides a convenient method to examine the diversity of organisms in nature and enables researchers to more comprehensively and accurately measure the relative abundance of various microorganisms in the biological environment (Chen C. K. et al., 2020). It is less labor-intensive and more efficient than previous culture-based techniques, and it provides deeper insights into the diversity of communities (Chen L. et al., 2020). A variety of fungi, such as *Alternaria, Fusarium,* and *Penicillium* among others, were mostly used in previous reports on Yuluxiang pear fungi based on culture technology (Hou et al., 2022). As far as we know, they only quantified the proportion of fungi that belonged to specific culturable taxa based on the media used. However, fungal diversity can be affected by differences in plant types, sample sizes, sampling time points, and extraction methods. Now, HTS has been utilized to study the community structure and microbial diversity on the surface of fruits and vegetables (Burgos et al., 2017), including apple (*Malus domestica*), grape (*Vitis vinifera*), lettuce (*Lactuca sativa*), corn meal, peels, peach (*Prunus persica*), pepper (*Capsicum* spp.), spinach (*Spinacea oleracea*), strawberry (*Fragaria* x *ananassa*), tomato (*Solanum lycopersicum*), and pear (*Pyrus* spp.) among others (Leff et al., 2013; Lopez-Velasco et al., 2013; Chen et al., 2019). In addition, it is widely used in agriculture, industry, medicine, and other fields (Salmaso et al., 2018; Zang et al., 2018).

Therefore, this study used Illumina MiSeq (Illumina, San Diego, CA, United States) next-generation sequencing technology to analyze the changes in fungal community structure and diversity at different stages after harvest of Yuluxiang pear, which laid a foundation for preventing postharvest disease and preserving Yulu pear, which is crucial to promoting the development of a circular economy in Yuluxiang pear.

## 2. Materials and methods

### 2.1. Materials

#### 2.1.1. Sample collection and preparation

Yuluxiang pears were harvested in Zhaizi Township, Xixian County, Linfen City, Shanxi Province, China, in September 2019. Fruits of uniform size that no obvious diseases, pests, or mechanical damage were selected. In addition, fruit with a soluble solids content of 12.5 to 16.1%, total sugar of 8.7 to 9.8%, and titratable acid of 0.08 to 0.17% were transported to the laboratory in a refrigerated truck within 24 h and stored in a cold storage facility at 4°C ± 0.5°C. Mixtures of the peels and pulp of pears that were stored in a cold storage at 4°C ± 0.5°C were cut into sections of approximately 1 cm × 1 cm every 30 d with a sterile knife and designated (A) for the initial 30 d sample (A), and then 60 d (B), 90 d (C), and 120 (D). Each treatment was conducted in triplicate.

### 2.2. Methods

#### 2.2.1. Determination of hardness, soluble solids, and titratable acid

The firmness of the pulp was measured with a GY-1 fruit firmness tester (Shandong, China). PAL-BX/ACIDF5 (Beijing, China) sugar and the integrated acid machine were used to determine the contents of titratable acid and soluble solids in the pulp.

#### 2.2.2. Genome extraction and PCR amplification

The samples were ground in liquid nitrogen to ensure uniformity. DNA kits (MoBio Laboratories, Inc., Carlsbad, CA, United States) were used to extract total DNA from the samples in the four storage periods. Simultaneously, a Nanodrop spectrophotometer (ND-1000; Thermo Fisher Scientific, Waltham, MA, United States) was utilized. The DNA was quantified, and the quality of DNA extraction was examined by 1.2% agarose gel electrophoresis (Zhao et al., 2020). The ribosomal ribonucleic acid (rRNA) gene ITS region using ITS1F (5’-CTTGGTCATAGAGAGTAA-3′) and ITS2 (5’-GCTGCGTTCTTCATC GATGC-3′) were amplified by PCR (Tang et al., 2022; Wang et al., 2022).

#### 2.2.3. Library construction and onboard sequencing

The sequencing libraries were created using Illumina’s TruSeq Nano DNA LT Library Prep Kit. The quality of library was tested on an analyzer with a DNA kit before sequencing. The qualified sequencing library was diluted in a gradient, mixed, and denatured by NaOH to single-stranded DNA for sequencing. The optimal length of the target fragment for sequencing was 200–450 bp.

#### 2.2.4. Diversity analysis

Diversity analyses of the community composition were conducted, including α-diversity and β-diversity. α-Diversity is primarily used to analyze the diversity and abundance of fungal communities in the samples (Xue et al., 2021). The Observed Species and Chao1 indices reflect the abundance of fungal flora between different timepoints of samples at low temperature, and the Shannon and Simpson indices reflect the diversity of fungi in each treatment. Studies of the α-diversity index also included dilution curves, a rarefaction curve, and coverage index to detect the degree of sequencing coverage of the operational taxonomic units (OTUs) (Zhang et al., 2019). The β-diversity analysis primarily included a principal coordinates analysis (PCoA) and a β-diversity index between-group difference analysis (Wang et al., 2019). The operational taxonomic unit (OTU) classification table primarily provided the distribution of OTU composition of each sample at the taxonomic level (phylum, class, order, family, genus, and species), which reflects the community structure of the models at different taxonomic levels (Wang et al., 2017).

#### 2.2.5. Data optimization and clustering

The sequences were optimized with Trimmomatic (v 0.4), Flash (v 1.2.11), USEARCH 10.0 and QIIME (v1.9.1) and a Perl program that was developed by Shanghai Yuanxin Biologics (Shanghai, China) that clustered the effective sequences according to 97% consistency that then became operational taxa. QIIME (v 1.9.1) and an RDP Classifier Bayesian algorithm performed classification analysis on 97% similar OTU representative sequences to calculate the index of fungal diversity. The R (v 4.0.3) language Vegdist and Hclust were used for community heatmap mapping, and Bray-Curtis distance calculations (Bray and Curtis, 1957) were used to conduct a cluster analysis. The R (v 4.0.3) language was used for the PCoA and PERMANOVA analyses. The relationship between fungal diversity and community structure and fruit hardness, titratable acid and soluble solids content was studied by a Spearman correlation analysis. The R language tool was used to create a correlation heatmap diagram.

#### 2.2.6. Statistical analysis

A statistical analysis of the test data was conducted using Microsoft Excel 2007 (Redmond, WA, United States) and SPSS 23.0 (IBM, Inc., Armonk, NY, United States), and Origin2021 (OriginLab, Northampton, MA, United States) was used to draw the horizontal distribution map of the phyla and genera. Duncan’s new complex range method was used to test the significance of difference.

## 3. Results

### 3.1. Determination of the hardness, total soluble solids, and titratable acid

The hardness, soluble solids, and titratable acid content of Yuluxiang pear fruits during storage are shown in Table 1. The fruit firmness, soluble solids, and titratable acid content decreased as the storage period was extended. In early storage, the fruit hardness decreased rapidly, and it then remained stable. The decrease in fruit hardness could be related to the gradual degradation of cellulose and the decomposition of pectin and starch in the fruit. In addition, the content of soluble solids also decreased as the time of storage was prolonged, which could be related to the gradual consumption of organic matter, such as soluble sugars, in the fruit that was converted into CO_2_ and H_2_O during the metabolic process. The titratable acid content decreased rapidly, and there were significant differences in different storage periods, which could be the result of ethanol absorption by the fruits that interacted with the organic acids (Table 1).

**Table 1:** Hardness, titratable acid, and solid soluble content of Yuluxiang pears in different storage periods.

Different treatments	Hardness/(kg·cm^−2^)	Total soluble solids content/%	Titratable acid content/%
30d	4.67 ± 0.47a	11.85 ± 0.02a	0.64 ± 0.03a
60d	4.43 ± 0.18a	11.77 ± 0.03ab	0.48 ± 0.04b
90d	4.43 ± 0.05a	11.61 ± 0.02bc	0.26 ± 0.05c
120d	4.38 ± 0.33a	11.5 ± 0.15c	0.13 ± 0.07d

Lowercase letters indicate significant differences between storage periods (*p* < 0.05).

### 3.2. Analyses of the diversity indices

A total of 1,792,803 valid sequences were obtained for total sequencing, with an average of 149,400 ± 16,282 sequences per sample. Based on the α-analysis of the sample, the rates of coverage of the 30 d(A), 60 d(B), 90 d(C) and 120 d(D) libraries were relatively high (>99.9%), indicating that sequencing increased the coverage of this species. The results were reliable, and the data met the sequencing requirements. Observed species and Chao1 were used to evaluate the abundance of fungal flora in different timepoints of samples at low temperature. Shannon and Simpson indices were used to assess the diversity of microorganisms in each treatment. The Observed species (78 ± 8.75) and Chao1 (78.33 ± 8.81) values at 30 d(A) were greater than those at 60 d(B), 90 d(C), and 120 d(D), indicating that the 30 d(A) treatment had the most fungi, followed by the 60 d(B), 90 d(C), and 120 d(D) groups. The Shannon and Simpson indices at 30 d(A) were 3.98 ± 0.04 and 0.089 ± 0.002, respectively, followed by lower values at 90 d(C), 60 d(B), and 120 d(D), indicating that the 30 d(A) treatment had the highest degree of fungal diversity. The Shannon and Simpson indices indicated there were specific differences in fungal diversity among different timepoints of samples at low temperature. Compared with other samples, the 30 d(A) samples exhibited a higher richness and diversity of fungi, indicating that there were more species in these samples, and the abundance and diversity of fungal flora in Yuluxiang pear changed with the storage time (Table 2).

**Table 2:** Alpha diversity index of different treatments.

Diversity index	Treatment			
	30d	60d	90d	120d
Chao1	78.33 ± 8.81	28 ± 7.79	24 ± 2.82	15.66 ± 1.69
Observed_species	78. ± 8.75	27 ± 8.24	22 ± 2.73	14 ± 1.79
Shannon index	3.98 ± 0.04	0.07 ± 0.03	0.32 ± 0.01	0.04 ± 0.02
Simpson index	0.089 ± 0.002	0.013 ± 0.006	0.112 ± 0.098	0.007 ± 0.005
Coverage	0.99999	0.99999	0.99999	0.99999

### 3.3. β-Diversity analysis

**Figure 1:**
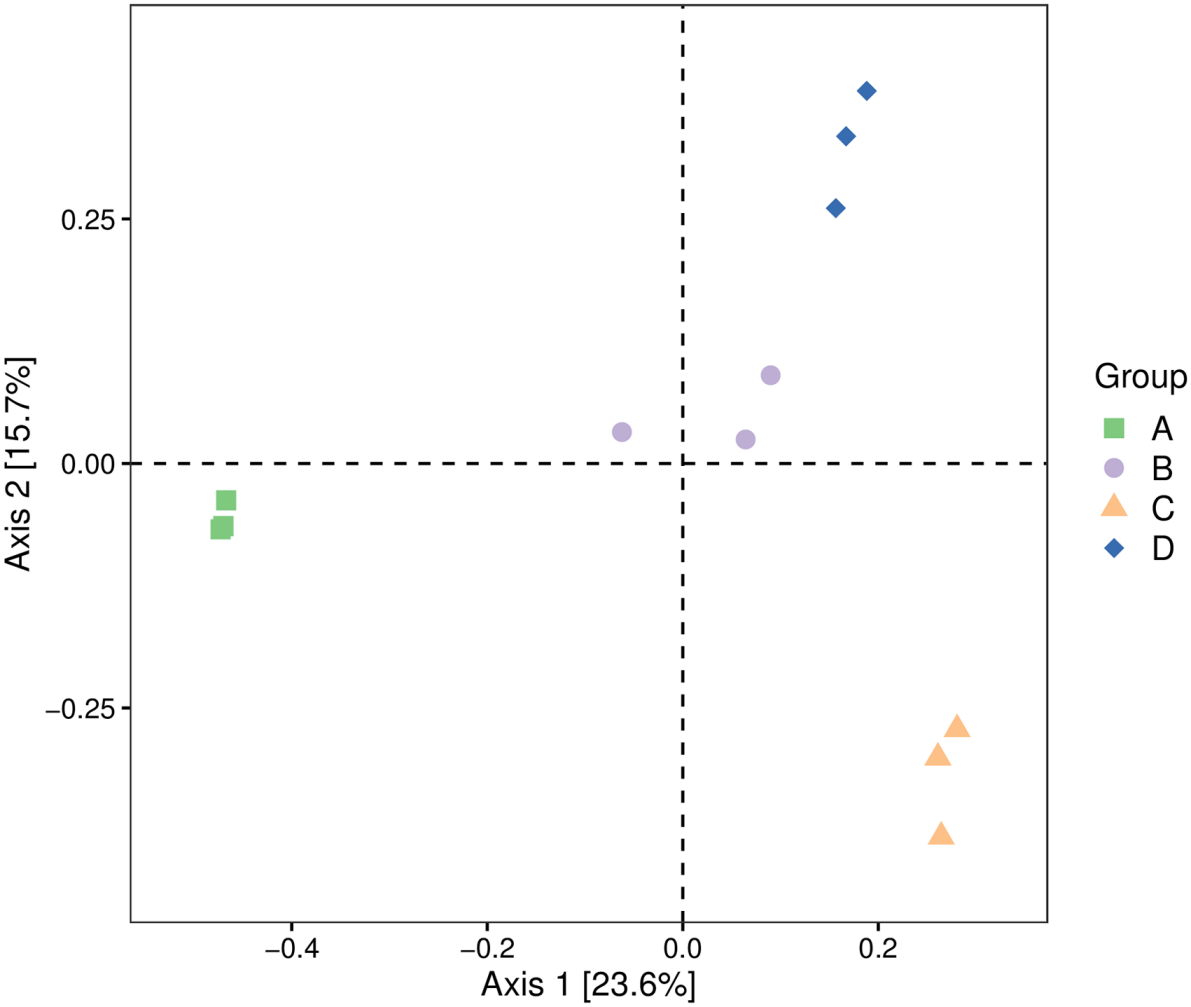
PCoA of fungal communities in Yuluxiang pears after storage at low temperatures for 30 d(A), 60 d(B), 90 d(C), and 120 d(D). Each point represents a sample, and the dots of different colors indicate different samples (groups). The percentages in the parentheses on the axis represent the proportion of sample difference data (distance matrix) that can be interpreted by the corresponding axis. PCoA, Principal coordinates analysis.

The fungal community composition of four groups of pear fruit samples was compared and analyzed. The diversity of four groups of pear fruit fungi was analyzed by a PCoA using the Jaccard distance algorithm to evaluate the similarity and difference of community composition. As shown in Figure 1, the fungal communities in the samples of the same treatment group were clustered, while the fungal communities in the samples of different treatment groups could be significantly separated, indicating that there were obvious differences between the fungi in the samples of different treatment groups. A PERMANOVA experimental analysis showed that the overall *p*-value was greater than 0.05, indicating that there were significant differences in OTU composition and relative abundance (Figure 1).

### 3.4. Venn diagram analysis of OTUs

**Figure 2:**
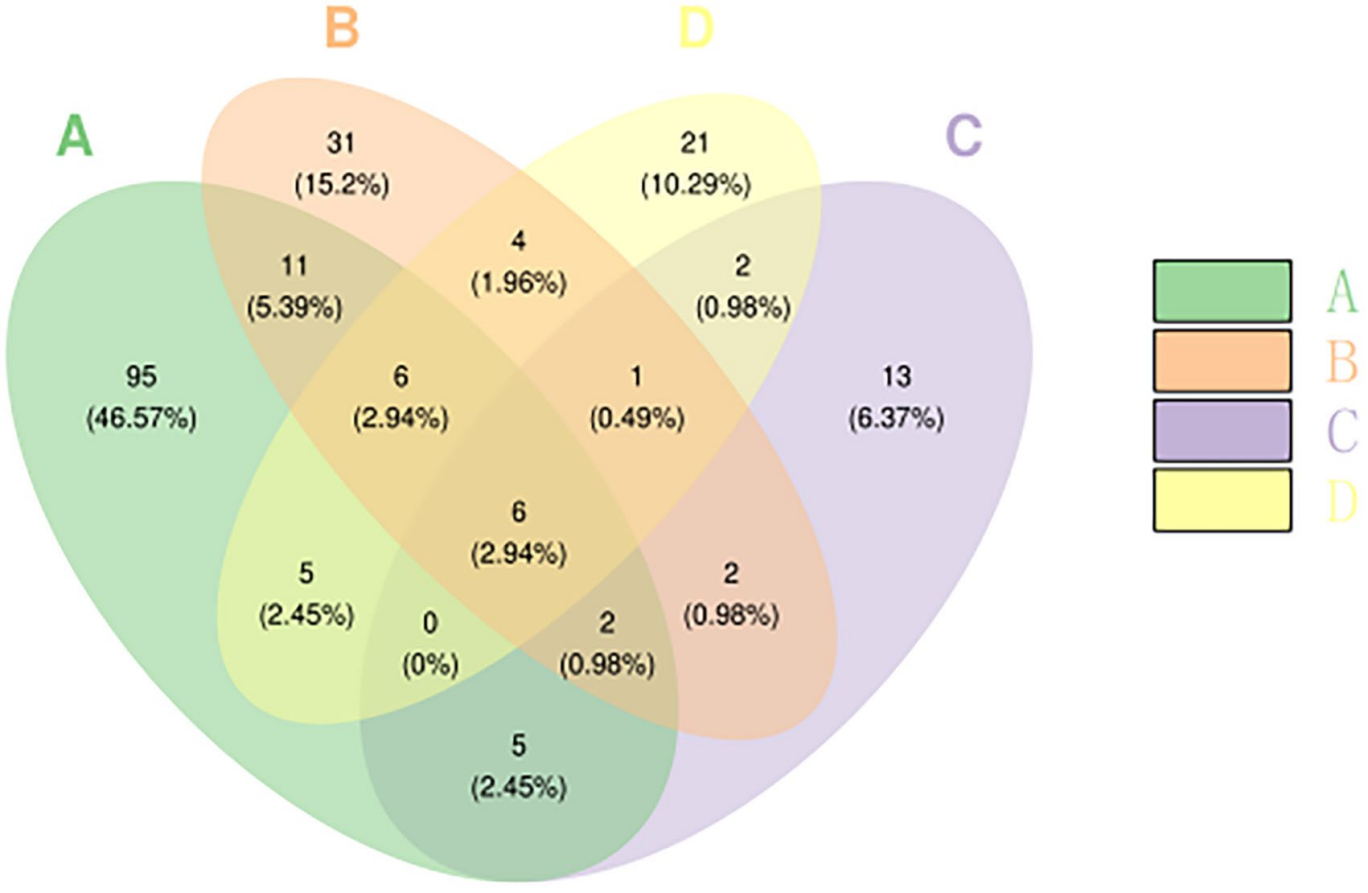
Microbial Venn plot of different samples based on the OTU level. Each ellipse represents a sample (group). The overlapping area between the ellipses indicates the common OTUs between the samples (group), and the number of each block indicates the number of OTUs contained in the block. OTU, operational taxonomic unit.

A Venn diagram can intuitively reflect the differences and similarities of the OTU composition of the fungal community in different Yuluxiang pear treatment groups. There were 46.57% (95) and 15.2% (31) of the specific fungal community OTU on 60 d(B), 6.37% (13) of the specific fungal community OTU on 90 d(C), and 10.29% of the 120 d(D) OTU (21). In addition, the 30 d(A), 60 d(B), 90 d(C), and 120 d(D) groups shared six OTUs (2.94%) (Figure 2). The unique OTUs of the 30 d(A) treatment were higher than those of the other groups, indicating that the fungal community after 30 d(A) treatment was more diverse than those in the 60 d(B), 90 d(C) and 120 d(D) groups.

### 3.5. Community composition analysis

**Figure 3:**
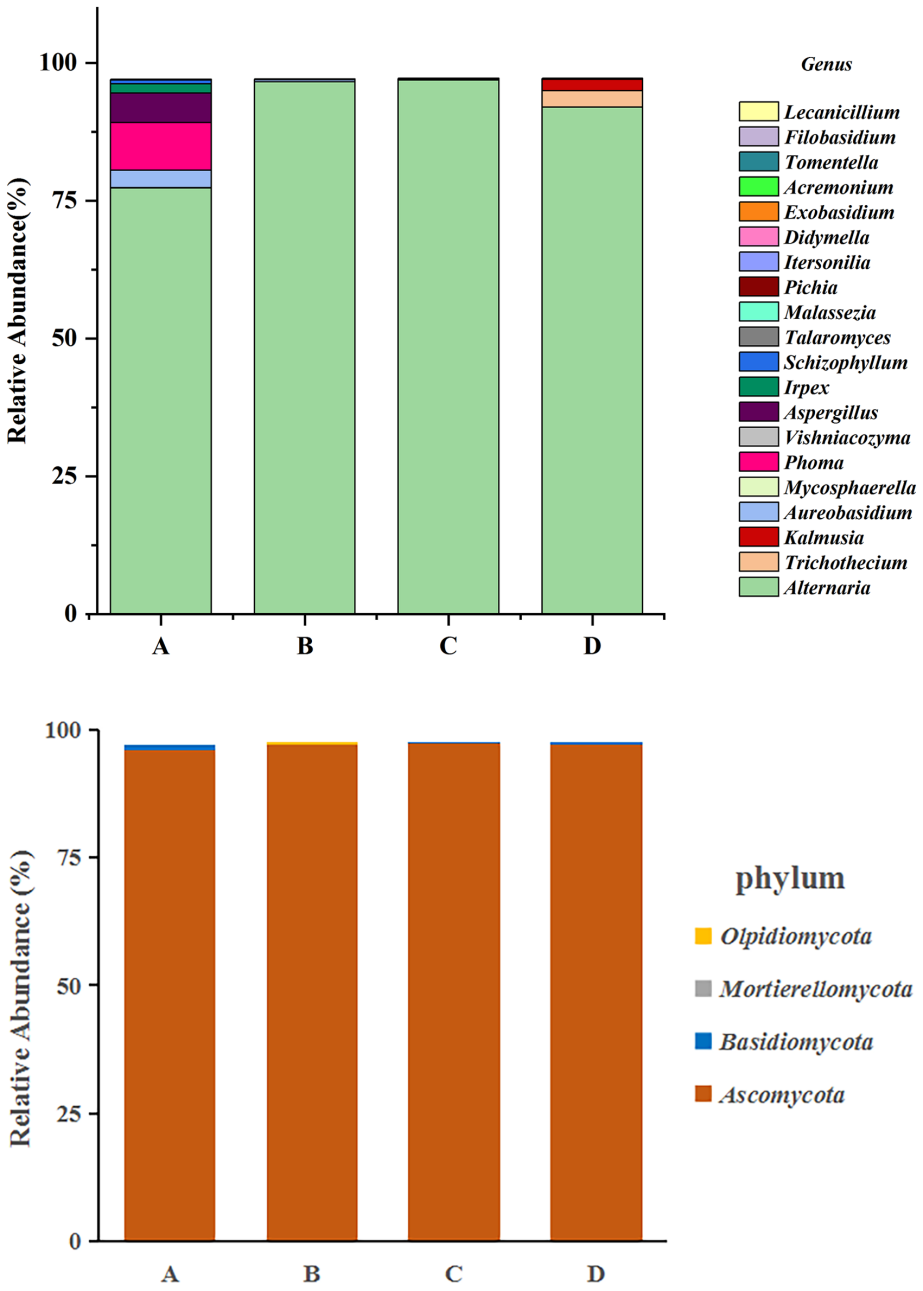
Analysis of fungal community diversity at the phylum level and genus level of Yuluxiang pears stored at low temperature for 30 d(A), 60 d(B), 90 d(C), 120 d(D), and different colored dots indicated different samples (groups).

According to the OTU annotation results of the four groups of samples of Yuluxiang pear, the fungi in the samples were identified as 6 phyla, 18 classes, 40 orders, 72 families, and 92 genera. The species diversity information in the samples was analyzed, and the histogram of the taxonomic abundance of the flora is shown in Figure 3. According to all the sequences in the library, the distribution of the four treatment groups of the Yuluxiang pear at the gate level is shown in Figure 3. The community distribution of the four treatment groups of Yuluxiang pear was primarily Ascomycota, and the abundance of Ascomycota in the four treatment groups was 96, 97, 97 and 97% at 30 d(A), 60 d(B), 90 d(C), and 120 d(D), respectively. The abundance of Ascomycota at the phylum level increased in parallel with the storage time. The 30 d(A) treatment was dominated by Ascomycota and Basidiomycota, with Basidiomycota accounting for 0.85%, while the abundances of Basidiomycota for the 60 d(B), 90 d(C) and 120 d(D) groups were 0.05, 0.05 and 0.14%, respectively. Basidiomycota in the 30 d(A) treatment group was more abundant than those in the 60 d(B), 90 d(C), and 120 d(D) groups. The abundance of Basidiomycota slowly decreased over time. The 30 d(A), 90 d(C), 120 d(D) groups did not contain Mortierellomycota or Olpidiomycota, and these phyla were present at relatively low abundance in the 60 d(B) group.

In terms of the horizontal composition of the genera, the relative abundances of Alternaria at 30 d(A), 60 d(B), 90 d(C) and 120 d(D) were 77.4, 96.6, 96.9 and 92%, respectively, and Alternaria was the dominant genus. In addition, the abundances of *Phoma*, *Aureobasidium, Aspergillus* and *Irpex* were 8.64, 3.2, 5.33 and 1.73% in the 30 d(A) treatment, and 0.39% in the 60 d(B) treatment. The abundance of *Mycosphaerella* in the 90 d(C) treatment was 0.26%, while it was 3.1% in the 120 d(D) treatment. The abundance of *Kalmusia* was 2.1%. As shown in Figure 3, *Alternaria* was the overwhelmingly dominant community in the four groups of samples. It was distributed across all the samples and dominant in most of them at the genus level. There was a gradual decline in the viability of *Phoma*, *Aureobasidium*, and *Aspergillus* in the 30 d(A), 60 d(B), 90 d(C), and 120 d(D) groups, which could have been owing to the poor culture conditions that hampered the growth of fungi. Thus, the viability of the fungi had been greatly reduced, or they even died. However, different pathogens, such as *Trichothecium* and *Kalmusia*, appeared later. They were present at relatively low levels (relative abundance<0.1%), so they were not analyzed.

### 3.6. Differential analysis of community structure among the microbiomes

**Figure 4:**
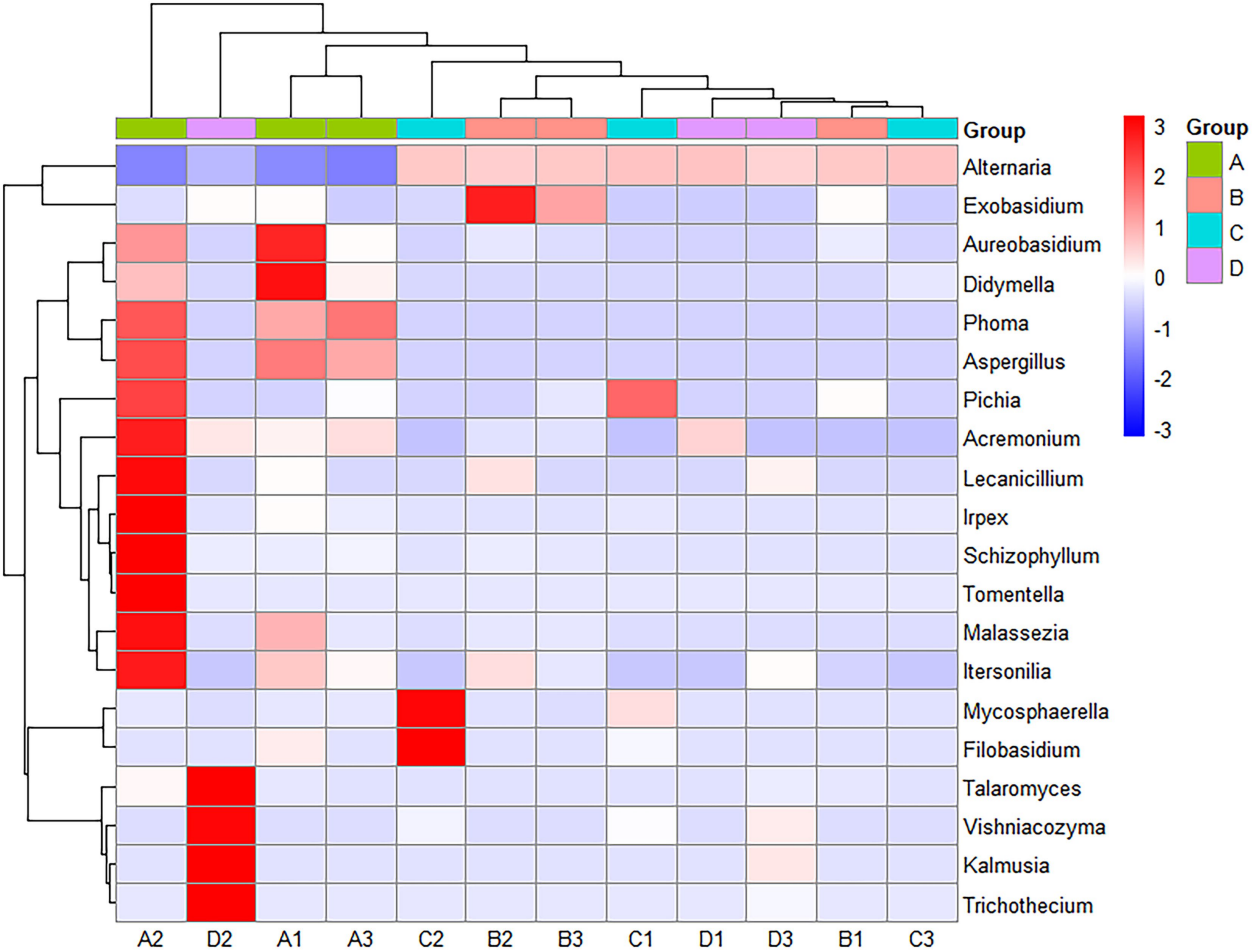
A cluster heatmap of the flora at the genus level. The red patch in the figure indicates that the genus is more abundant in this sample than in the other samples, and the blue patch indicates that the genus is less abundant in this sample than in the other samples.

**Figure 5:**
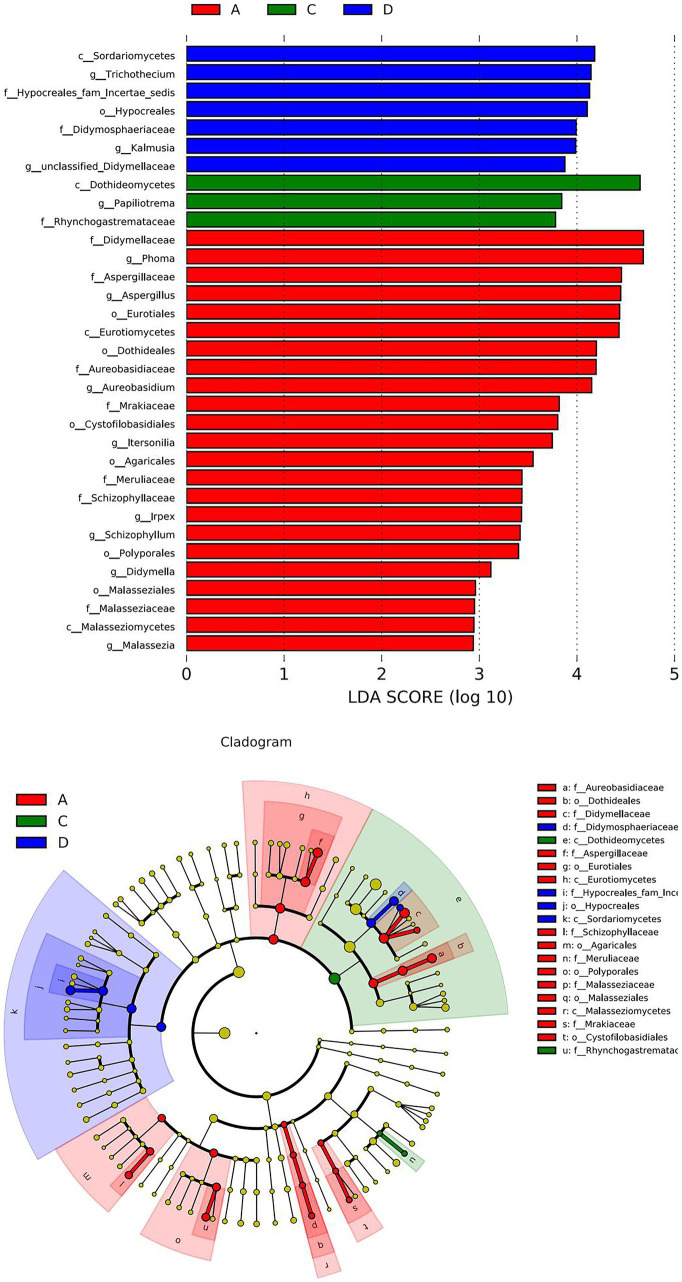
Histogram of the LDA effect values of marker species and differential taxa between groups. The LDA ordinate is a taxa with significant differences between the groups, and the abscissa visually displays the logarithmic score values of the LDA analysis for each taxa in a bar chart. The taxonomic clade shows the taxonomic hierarchical relationships of the main taxa in the sample community from the phylum to genus (from inner circle to outer circle). The node size corresponds to the average relative abundance of that taxa. Hollow nodes represent taxa with insignificant intergroup differences, while the nodes of other colors indicate that these taxa exhibit significant between-group differences and are more abundant in the grouped sample represented by that color. Letters identify the names of taxa that differ significantly between groups. LDA, linear discriminant analysis.

Figure 4 shows a heatmap analysis of the distribution of the 20 OTUs with the highest abundance between groups. A heatmap can visually reflect the similarity and difference of the fungal community composition of each group through color changes, which is more convenient to find genera that were more or less distributed in the sample. A redder color indicates that a higher proportion of the genus was contained in the sample. A bluer color indicates a lower proportion. A lateral homogenization treatment was conducted. As shown in Figure 4, A1 and A3 were clustered in A; B1 and B3 were clustered in B, and D1 and D3 were clustered in D. The similarity of their microbiota was relatively higher than those of the other samples in the same group. However, C1, C2 and C3 were separated in the C treatment, indicating that there were differences between the fungal communities in the same batch of Yuluxiang pear, and there were also differences between the fungal communities of different batches. In addition, LDA histograms (default threshold: LDA > 2) and evolutionary clade plots (Figure 5) were outputted to use LEfSe to identify the major groups that were specific between the groups. There was no differentiation of species in the 60 d(B) samples.

The LDA histogram shows the fungal microorganisms with significant differences in 30 d(A), 90 d(C), and 120 d(D) of the sample, and a longer bar for the taxa indicated that they were more significant. The color of the bar chart indicated that the group of samples with the highest abundance corresponded to the taxon. *c_Sordariomycetes, g_Trichothecium, f_Hypocreales_fam_Incertae_sedis, o_Hypocrea les, f_Didymosphaeriaceae, g_Kalmusia* and *g_unclassified_Didymellaceae* were more prevalent at 120 d(D) than in 30 d(A) and 60 d(B). There was a significant difference in 90 d(C). The *c_Dothideomycetes, g_Papiliotrema* and *f_Rhyncho gastremataceae* in 90 d(C) were significantly different from those in the 30 d(A), 60 d(B) and 120 d(D) groups. *f_Didymellaceae, g_Phoma, f_Aspergillus, o_Eurotiales, c_Eurotiomycetes, o_Dothideales, g_Aureobasidium, f_Mrakiaceae, o_Cystofilobasidiales, g_Itersonilia, o_Agaricales, f_ in 30D Meruliaceae, g_Irpex, g_Schizophyllum, o_Polyporales, g_Didymella* and *g_Malassezia* were significantly different from those in the 60 d(B), 90 d(C) and 120 d(D) groups.

### 3.7. Correlation of fruit hardness, titratable acid, and solid solubility content with the fungal community structure

**Figure 6:**
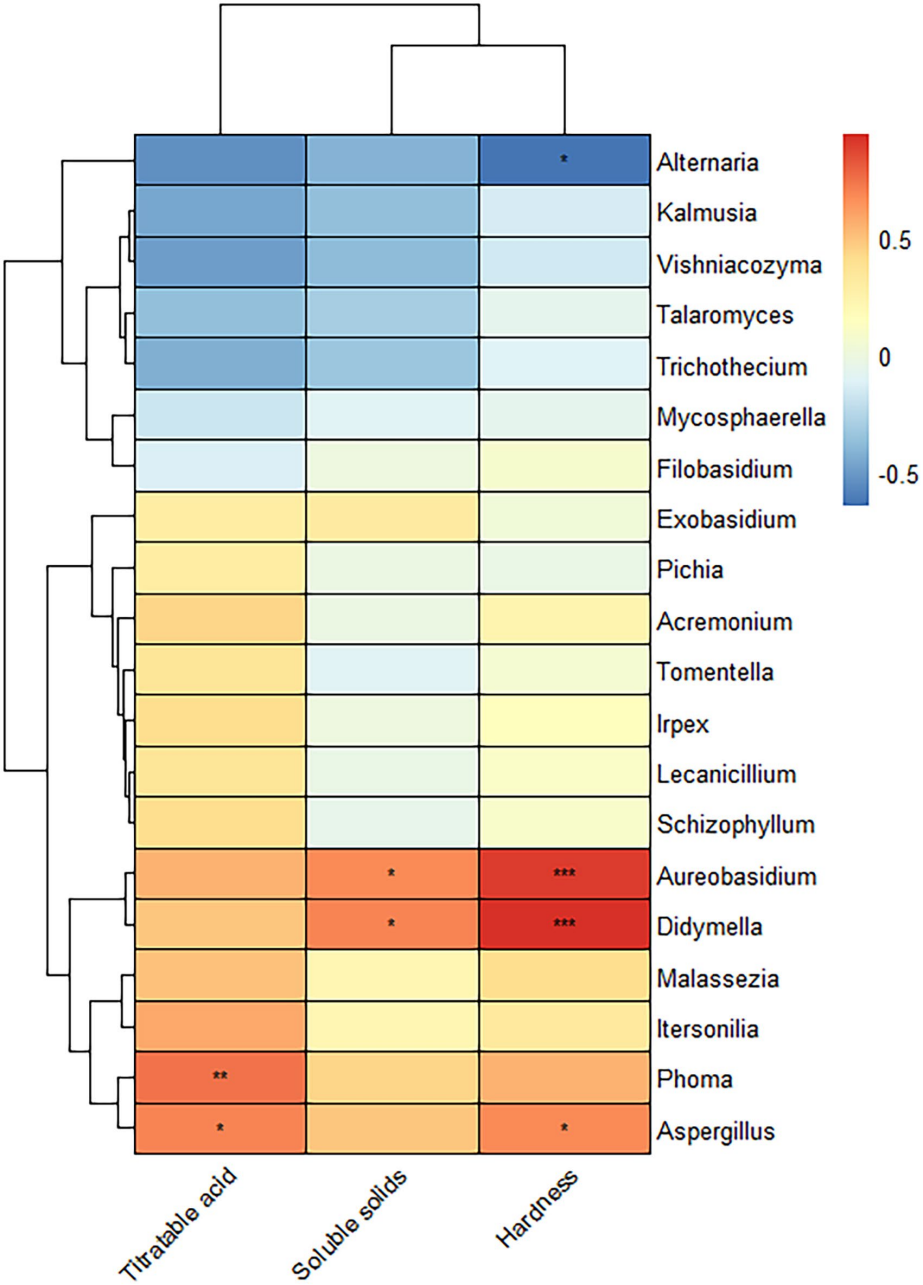
Correlation analysis of the fungi of Yuluxiang pear with fruit hardness, titratable acid, and soluble solids in different storage periods. Each column represents a bacterium. Blue is a negative correlation, and red is a positive correlation. * is a distinctive identifier.

Figure 6 is a heatmap created by a correlation analysis of the 20 OTUs with the highest levels of genera and fruit hardness, titratable acid and soluble solids. *Aureobasidium* and *Didymella* positively correlated with the soluble solids and hardness. *Phoma* positively correlated with the titratable acid, and *Aspergillus* positively correlated with titratable acid and hardness.

## 4. Discussion

In China, pears are harvested in season, and most of them are stored refrigerated for long-term sales. During the past 10 years, the implementation of refrigerated transportation has extended the postharvest storage of pears and their shelf life. However, with the extension of the storage period, pears will have some diseases owing to the invasion of fungi. From a general perspective, cold storage is often thought to decrease the fruit ripening process and delay the growth of spoilage fungi. However, some plant pathogenic fungi can cause postharvest deterioration, which seriously affects the safety and quality of fruits (Prasanna et al., 2007; Morales et al., 2010; Shen et al., 2018). Therefore, identifying the fungal diversity of pear fruits has become an important object of fruit research, which can provide a new perspective to predict and control postharvest diseases in pear fruits.

In this study, HST was used to analyze the structure and diversity of fungal colonization of Yuluxiang pear fruits in different storage periods. The sparsity curve suggests that the current sequencing depth is deep enough to detect most of the fungi on pears.

The comparison of the three biodiversity indices of Chao, Shannon, and Simpson revealed that the highest abundance of flora and fungal diversity was observed after 30 d(A) of treatment. A ß-diversity analysis showed that the fungal communities in the samples of the same treatment group were clustered together. The fungal communities in the samples of different treatment groups could be significantly separated, indicating that there were clear differences in fungi between the samples of different treatment groups. Furthermore, low temperature can delay the growth of spoilage pathogens, and the effect of storage time on the fungal community is complex (Dadzie et al., 2021; Tian et al., 2021). The results showed that cryogenic storage reduced the α-diversity of microorganisms over time. It is hypothesized that some fungi will become intolerant with prolonged storage, inhibiting their growth.

In the community structure analysis of the Yuluxiang pear fruit fungi at different storage stages, it emerged that the level of composition of fungi at the phylum level was relatively simple and was primarily composed of Ascomycota, Basidiomycota, and Mortierellomycota. Among them, Ascomycota dominated the number of community compositions. There were differences in the level of Basidiomycota among the groups at 30 d(A), 60 d(B), and 90 d(C), presumably owing to the colonization of pathogenic fungi. HST was used to comprehensively analyze the samples, and Ascomycota was the primary phylum that was detected. It is the largest phylum in the fungal kingdom and was present in all the samples (Abdelfattah et al., 2016; Shen et al., 2018). There are studies that have shown that the main fungi on the surface of pear are Ascomycota, which grow quickly and can survive harsh conditions with low nutrient levels (Challacombe et al., 2019). They can adapt to a wide range of substrates in challenging environments, such as ultraviolet light, water and high temperature stress (Jaber et al., 2012). At the level of genus, *Alternaria* was more abundant in all the different timepoints of samples at low temperature. In addition, Hou et al. (2022) obtained *Alternaria* during the isolation and identification of postharvest pathogenic fungi of Yuluxiang pear, indicating that *Alternaria* is the dominant genus that causes postharvest disease on Yuluxiang pear. *Alternaria alternata* is highly adaptable to Yuluxiang pears and is also the primary pathogenic fungus that causes pear black spot disease in China (Roberts, 2005; Wang and Zhang, 2010; Ttpabc et al., 2019). As shown in the figure, fungal diversity at the genus level slowly stabilized, and studies have shown that after harvesting, the fungal microbiome changes significantly during storage and becomes an aging plant microbiome. It is characterized by a decrease in the diversity of fungal microbial diversity and an increase in the degradation and decomposition of the special fungal microflora (Kusstatscher et al., 2020). A LEfSe analysis yielded species that varied between the different sample groups. As compared with the other three groups of different species, the 30 d(A) treatment had the highest abundance of fungi. The results indicate that the fungal community structure is the most complex during the early stages of preservation of Yuluxiang pear. With the extension of storage time, the fungal community structure was relatively stable.

Temperature, relative humidity, pH, soluble solids, and hardness are the main factors that influence fungal colonization (Li et al., 2022). The results of this study on the correlation between fungal communities and fruit quality illustrate the hardness, titratable acid, and solid soluble content of Yuluxiang pears at different postharvest storage periods. A downward trend was observed, which was similar to the findings of Zhang et al. (2021). The results of the study on the correlation between fungal communities and fruit quality indicated that *Aureobasidium* and *Didymella* increased when the content of soluble solids and hardness increased. Similarly, the abundances of *Aureobasidium* and *Didymella* also increased when the content of soluble solids and hardness increased. The increase in *Phoma* correlated with the fruit titratable acid. The fruit titratable acid and hardness increased in parallel with the abundance of *Aspergillus*, *Aureobasidium*, *Phoma*, and *Aspergillus*, which were all abundant at 30 d(A), indicating that the fungal diversity of the sample at 30 d(A) was closely related to fruit hardness, titratable acid and soluble solids.

## 5. Conclusion

HTS was used to analyze the community structure and fungal diversity of Yuluxiang Pear at different storage periods. The diversity of treated microflora was found to differ as the storage time was extended. Compared with the treatment at the storage stage, the composition of the fungal community was significantly different from that at the beginning of storage. Comparison of the fungal phyla showed that Ascomycota was the dominant phylum in different treatment groups, with *Alternaria* dominating at the genus level. In addition, the relationship between fungal community structure and hardness, titratable acid and soluble solids at different storage stages was also discussed. To improve the prevention and control of fruit diseases and quality control of Yuluxiang pear, this study provides assistance for the industrial production of Yulu pear.

## Data availability statement

The datasets presented in this study can be found in online repositories. The names of the repository/repositories and accession number(s) can be found at: https://www.ncbi.nlm.nih.gov/, PRJNA909451.

## Author contributions

All authors listed have made a substantial, direct, and intellectual contribution to the work and approved it for publication.

## Funding

This work was supported by the Natural Science Foundation of Shanxi Province (201901D111451) and the Research Innovation Team Training Project of Shanxi Academy of Agricultural Sciences (YGC2019TD05).

## Conflict of interest

The authors declare that the research was conducted in the absence of any commercial or financial relationships that could be construed as a potential conflict of interest.

## Publisher’s note

All claims expressed in this article are solely those of the authors and do not necessarily represent those of their affiliated organizations, or those of the publisher, the editors and the reviewers. Any product that may be evaluated in this article, or claim that may be made by its manufacturer, is not guaranteed or endorsed by the publisher.

## References

[ref1] Abdelfattah A. Wisniewski M. Droby S. Schena L. (2016). Spatial and compositional Vari ation in the fungal communities of organic and conventionally grown apple fruit at the consumer point-of-purchase. Hort. Res. 3:16047. doi: 10.1038/HORTRES.2016.47 PMC5051542 27766161

[ref2] Abdel-Wahab M. A. Bahkali A. H. Elgorban A. M. Jones E. B. (2021). High-throughput amplicon sequencing of fungi and microbial eukaryotes associated with the seagrass Halophila stipulacea (Forssk.) Asch. From Al-Leith mangroves, Saudi Arabia. Mycol. Prog. 20, 1365–1381. doi: 10.1007/S11557-021-01744-2

[ref110] Bray J. R. Curtis J. T. (1957). An ordination of the upland forest communities of southern Wisconsin. Ecological monographs 27, 326–349. doi: 10.2307/1942268

[ref3] Burgos M. Aguayo M. Pulido R. P. Gálvez A. Lucas R. (2017). Analysis of the bacterial biodiversity of peaches under refrigerated storage after treatment by high hydrostatic pressure. Food Bioprod. Process. 102, 55–61. doi: 10.1016/j.fbp.2016.12.003

[ref4] Challacombe J. F. Hesse C. N. Bramer L. M. McCue L. A. Kuske C. R. (2019). Genomes and secretomes of Ascomycota fungi reveal diverse functions in plant biomass decomposition and pathogenesis. BMC Genomics 20:976. doi: 10.1186/s12864-019-6358-x, PMID: 31830917 PMC6909477

[ref5] Chen L. Li Y. Z. Jin L. He L. Ao X. L. Liu S. L.. (2020). Analyzing bacterial community in pit mud of Yibin baijiu in China using high throughput sequencing. Peer J. 8:e9122. doi: 10.7717/peerj.9122, PMID: 32435541 PMC7227652

[ref6] Chen J. R. Yan R. X. Hu Y. F. Zhang N. Hu H. Y. (2019). Compositional shifts in the fungal diversity of garlic scapes during postharvest transportation and cold storage. LWT 115:108453. doi: 10.1016/j.lwt.2019.108453

[ref7] Chen C. K. Zhang H. J. Zhang X. J. Dong C. H. Xue W. T. Xu W. T. (2020). The effect of different doses of ozone treatments on the postharvest quality and biodiversity of cantaloupes. Postharvest Biol. Technol. 163:111124. doi: 10.1016/j.postharvbio.2020.111124

[ref8] Dadzie R. G. Amoah R. S. Ampofo-Asiama J. Quaye B. Kizzie-Hayford N. Abano E. E. (2021). Improving the storage quality of eggplants (solanum Aethiopicum L.) fruit using aloe Vera gel coating. J. Food Technol. Res. 8, 58–66. doi: 10.18488/journal.58.2021.82.58.66

[ref9] Dai C. W. Xu Y. Wu C. J. Cai B. (2019). Application progress in food-borne pathogens detection based on high-throughput sequencing technology. J. Food Safe. Qual. 10, 6006–6012. doi: 10.19812/j.cnki.jfsq11-5956/ts.2019.18.007

[ref10] Hou Y. R. Gao Z. F. Yang Z. G. Chen T. Zhang Y. Guan J. F.. (2022). Isolation, identification and biological characteristics of postharvest pathogens of Yuluxiang. Pear. Sci. Technol. Food Ind. 43, 122–129. doi: 10.13386/j.issn1002-0306.2021110364

[ref11] Jaber B. M. Al-Silawi R. Al-Najjar T. (2012). Isolation and molecular identification of ascomycetes in sediments and waters of the Gulf of Aqaba. Red Sea. Natural ence 04, 555–561. doi: 10.4236/ns.2012.48074

[ref12] Kusstatscher P. Cernava T. Abdelfattah A. Gokul J. Korsten L. Berg G. (2020). Microbio me approaches provide the key to biologically control postharvest pathogens and storability of fruits and vegetables. FEMS Microbiol. Ecol. 96:fiaa119. doi: 10.1093/femsec/fiaa119, PMID: 32542314

[ref13] Leff J. W. Noah F. Gabriele B. (2013). Bacterial communities associated with the surfaces of fresh fruits and vegetables. PLoS One 8:e59310. doi: 10.1371/journal.pone.0059310, PMID: 23544058 PMC3609859

[ref14] Li H. Zhang Y. Gao C. Gao Q. Cheng Y. Zhao M.. (2022). Mycotoxin production and the relationship between microbial diversity and mycotoxins in Pyrus bretschneideri Rehd cv. Huangguan Pear. Toxins. 14:699. doi: 10.3390/toxins14100699, PMID: 36287968 PMC9610726

[ref15] Li W. H. Zhang H. Y. Li P. X. Tibiru A. M. Yang Q. Y. Peng Y. P.. (2016). Biocontrol of postharvest green mold of oranges by hanseniaspora uvarum Y3 in combination with phosphatidylc holine. Biol. Control 103, 30–38. doi: 10.1016/j.biocontrol.2016.07.014

[ref16] Lopez-Velasco G. Carder P. A. Welbaum G. E. Ponder M. A. (2013). Diversity of the spin ach (Spinacia oleracea) spermosphere and phyllosphere bacterial communities. FEMS Microbiol. Lett. 346, 146–154. doi: 10.1111/1574-6968.12216, PMID: 23859062

[ref17] Ma F. L. Du Y. M. Wang Y. Tong W. Liu B. L. Wang W. H.. (2019). Effect of 1-Methylcyclopropene(1-MCP) on quality and chlorophyll maintenance of postharvest 'Yuluxiang' pear. Acta Horticult. Sin. 46, 2299–2308. doi: 10.16420/j.issn.0513-353x.2018-0676

[ref18] Morales H. S. Marín Ramos A. J. Sanchis V. (2010). Influence of post-harvest technologies applied during cold storage of apples in Penicillium expansum growth and patulin accumulation: a review. Food Control 21, 953–962. doi: 10.1016/j.foodcont.2009.12.016

[ref19] Prasanna V. Prabha T. N. Tharanathan R. N. (2007). Fruit ripening phenomena–an overview. Crit. Rev. Food Sci. Nutr. 47, 1–19. doi: 10.1080/10408390600976841, PMID: 17364693

[ref20] Roberts R. G. (2005). Alternaria yaliinficiens sp. nov. on Ya Li pear fruit: from interception to iden tification. Plant Dis. 89, 134–145. doi: 10.1094/PD-89-0134, PMID: 30795215

[ref21] Salmaso N. Albanese D. Capelli C. Boscaini A. Pindo M. Donati C. (2018). Diversity and cyclical seasonal transitions in the bacterial community in a large and deep perialpine lake. Microb. Ecol. 76, 125–143. doi: 10.1007/s00248-017-1120-x, PMID: 29192335

[ref22] Shen Y. Nie J. Dong Y. Kuang L. Li Y. Zhang J. (2018). Compositional shifts in the surface fungal communities of apple fruits during cold storage. Postharvest Biol. Technol. 144, 55–62. doi: 10.1016/j.postharvbio.2018.05.005

[ref23] Shen Y. M. Nie J. Y. Li Z. X. Li H. F. Wu Y. L. Dong Y. F.. (2018). Differentiated surface fungal communities at point of harvest on apple fruits from rural and peri-urban orchards. Sci. Rep. 8, 2165–2112. doi: 10.1038/s41598-017-17436-5, PMID: 29391402 PMC5794916

[ref24] Sun P. P. Cui J. C. Jia X. H. Wang W. H. (2017). Complete genome sequence of bacillus velezensis l-1, which has antagonistic activity against pear diseases. Genome Announc. 5:e01271. doi: 10.1128/genomeA.01271-17, PMID: 29192072 PMC5722058

[ref25] Tang L. Wei W. L. Zhao Y. J. Yu W. P. Wu Z. Y. Zeng L.. (2022). Analysis of physicochemical characteristics and fungal community diversity during fermentation of industrial radish kimchi. Food Ferment. Ind. 48, 25–31. doi: 10.13995/j.cnki.11-1802/ts.028275

[ref26] Tian F. Cai E. W. Kou X. H. Fan G. J. Li T. (2021). Surface fungal community diversity change and potential pathogens of Ginkgo biloba seed during cold storage. Food Biosci. 41:100981. doi: 10.1016/j.fbio.2021.100981

[ref27] Ttpabc E. Ec E. Dwsabc D. Jp E. Hpab C. (2019). Pathogenetic process monitoring and early detection of pear black spot disease caused by alternaria alternata using hyperspectral imaging. Postharvest Biol. Technol. 154, 96–104. doi: 10.1016/j.postharvbio.2019.04.005

[ref28] Wang X. D. Ban S. D. Hu B. D. Qiu S. Y. Zhou H. X. (2017). Bacterial diversity of Moutai-flavour Daqu based on high-throughput sequencing method. J. Inst. Brew. 123, 138–143. doi: 10.1002/jib.391

[ref29] Wang B. T. Yang W. L. Lei G. Li S. S. Liu D. H. (2022). Analysis of fungal diversity in rhizosphere soil of konjac based on high-throughput sequencing. Southwest China J. Agric. Sci. 35, 804–811. doi: 10.16213/j.cnki.scjas.2022.4.010

[ref30] Wang Y. B. Zhang Y. X. (2010). Salicylic acid induces the accumulation of defense-related enzym es in Whangkeumbae pear and protects from pear black spot. Front. Agric. China 4, 215–219. doi: 10.1007/s11703-010-0002-5

[ref31] Wang M. Y. Zhao Q. S. Su C. Yang J. G. (2019). Analysis of the microbial community structure during brewing of Sichuan Xiaoqu baijiu. J. Am. Soc. Brew. Chem. 77, 210–219. doi: 10.1080/03610470.2019.1605033

[ref32] Xu S. Lu W. J. Liu Y. T. Ming Z. Y. Liu Y. J. Meng R. H.. (2016). Structure and div ersity of bacterial communities in two large sanitary landfills in China as revealed by high-throughput sequencing (miseq). Waste Manag. 63, 41–48. doi: 10.1016/j.wasman.2016.07.047 27515184

[ref33] Xue B. Yu J. J. Zhang J. C. Hao F. Zhang X. M. Dong J. H.. (2021). Microbial diversity analysis of vineyard son the eastern foothills of the Helan Mountain region using high-th roughput sequencing. Food Sci. Technol. 42:66320. doi: 10.1590/fst.66320

[ref34] Yu T. Chen Y. Lu H. Zunun M. Chen F. Tao Z.. (2012). Effect of cryptococcus laurentii and calcium chloride on control of penicillium expansum and botrytis cinerea infections in pear fruit. Biol. Control 61, 169–175. doi: 10.1016/j.biocontrol.2012.01.012

[ref35] Zang J. H. Xu Y. S. Xia W. S. Yu D. W. Gao P. Jiang Q. X.. (2018). Dynamics and diversity of microbial community succession during fermentation of suan yu, a chinese traditional fermented fish, determined by high throughput sequencing. Food Res. Int. 111, 565–573. doi: 10.1016/j.foodres.2018.05.076, PMID: 30007719

[ref36] Zhang S. W. Chen X. Zhong Q. D. Zhuang X. L. Bai Z. H. (2019). Microbial community analyses associated with nine varieties of wine grape carposphere based on high-throughput sequencin g. Microorganisms. 7:668. doi: 10.3390/microorganisms7120668, PMID: 31835425 PMC6956142

[ref37] Zhang Q. Shi W. C. Zhou B. Du H. Y. Xi L. Q. Zou M.. (2020). Variable characteristics of microbial communities on the surface of sweet cherries under different storage conditions. Postharvest Biol. Technol. 173:111408. doi: 10.1016/j.postharvbio.2020.111408

[ref38] Zhang W. Zhao Y. L. Yang Z. G. Wang L. (2021). Effect of different temperature storage on fruit Q uality and chlorophyll fluorescence parameters of Yuluxiang pears in Xixian County. J. Shanxi Agricul. Sci. 49, 236–242. doi: 10.3969/j.issn.1002-2481.2021.02.23

[ref39] Zhao X. X. Wang Y. R. Cai W. C. Yang M. J. Zhong X. D. Guo Z.. (2020). High-throughput sequencing-based analysis of microbial diversity in rice wine koji from different areas. Curr. Microbiol. 77, 882–889. doi: 10.1007/s00284-020-01877-9, PMID: 31950235

